# Impact of Mediterranean Diet Pattern Adherence on the Physical Component of Health-Related Quality of Life in Middle-Aged and Older Active Adults

**DOI:** 10.3390/nu16223877

**Published:** 2024-11-13

**Authors:** Javier Conde-Pipó, Antonio Martinez-Amat, Agustín Mora-Fernández, Miguel Mariscal-Arcas

**Affiliations:** 1 Department of Health Sciences, Faculty of Health Sciences, University of Jaén, 23071 Jaén, Spain; jconde@ujaen.es (J.C.-P.); amamat@ujaen.es (A.M.-A.); 2 Health Science and Nutrition Research (HSNR-CTS1118), Department of Nutrition and Food Science, School of Pharmacy, University of Granada, 18071 Granada, Spain; agusmora@correo.ugr.es; 3 Instituto de Investigación Biosanitaria de Granada (IBS.GRANADA), 18012 Granada, Spain

**Keywords:** mediterranean diet, health-related quality of life, older adult, healthy ageing, active life

## Abstract

**Background/Objectives**: The Mediterranean dietary pattern (MedDiet) has numerous health benefits, particularly in preventing chronic diseases and improving well-being. Given the ageing population, understanding its impact on older adults’ physical health is essential. This study examines how adherence to the MedDiet influences the physical component (Comp-p) of health-related quality of life (HRQoL) across various age groups, providing insights for tailored dietary interventions. **Methods**: A cross-sectional study was conducted with active adults aged 41–80, categorised into four age groups (41–50, *n* = 116; 51–60, *n* = 225; 61–70, *n* = 135; 71–80, *n* = 44). Data were collected using the SF-36 and MEDAS questionnaires. Com-p scores were analysed based on MedDiet adherence (poor or good) and age. **Results**: In the 71–80 age group, a significant correlation was found between Comp-P and MedDiet adherence (r = 0.367, *p* = 0.014), with significantly higher Com-P scores in the good adherence group (50.10 ± 7.39) compared to the poor group (44.46 ± 7.73; *p* = 0.015; d = 0.74). The loss of adherence to the Mediterranean diet in this age group was attributed to low consumption of vegetables (36.36%), tree nuts (47.73%), legumes (50.00%), fish (52.27%), and fruit (56.82%). **Conclusions**: In individuals aged 71–80, lower adherence to the Mediterranean diet is associated with a decline in self-perceived physical health, attributed to the reduced intake of fresh vegetables, legumes, fish, and fruit. These findings emphasise the importance of promoting Mediterranean dietary adherence in later life to maintain optimal physical well-being.

## 1. Introduction

The increase in life expectancy presents a significant challenge for society, which must address increasing healthcare costs, and for individuals, who must take measures to prevent disability or chronic illness to maintain independence and quality of life [1]. Health-related quality of life (HRQoL) is often used as a key indicator for monitoring population health [2], as even minor subjective changes in physical or mental health can be highly significant for many older adults with one or more chronic conditions [3]. HRQoL is a multidimensional construct that reflects an individual’s self-perceived mental and physical health in relation to their overall quality of life [4,5]. Improving HRQoL is a primary goal of healthcare systems during the ageing process, as it provides valuable health insights into both individual and population levels [6]. It is frequently assessed using self-reported questionnaires such as the SF-36 or its short version, the SF-12 [7].

The physical component of HRQoL, which includes physical health and physical functioning, tends to decline with age due to changes in skeletal muscle and body composition [1]. These changes lead to reduced functional ability and limitations in performing activities of daily living [5,7]. Most research on HRQoL has primarily focused on the role of physical activity (PA) habits, rather than dietary patterns. Nevertheless, a proper nutritional status plays a critical role in the prevention or delay of ageing-related disease [8]. An unhealthy diet impairs physiological function and increases disease risk while improving diet is key to enhancing immune, cognitive, and overall physiological health [9].

The Mediterranean diet (MedDiet) was first described by Ancel Keys in the 1960s as a diet low in saturated fat and rich in vegetable oils, characteristic of Greece and southern Italy [10]. This dietary pattern is commonly defined by high intakes of extra-virgin olive oil, leafy green vegetables, fruits, cereals, nuts, and legumes, along with moderate consumption of fish, other meats, dairy products, and red wine, and limited intake of eggs and sweets [10]. According to the scientific literature, the MedDiet includes three to nine servings of vegetables, one to two servings of fruit, one to thirteen servings of cereals, and 1.5 to 8 servings of olive oil per day [10]. Its approximate energy content is 9.3 MJ/day, with around 37% of energy from total fat (19% from monounsaturated fatty acids (MUFAs), 5% from polyunsaturated fatty acids (PUFAs), and 9% from saturated fatty acids (SFAs)), 15% from protein, and 43% from carbohydrates [10]. These characteristics, together with a relatively high average flavonoid intake of approximately 344.9 mg per day and ease of adherence, particularly among older adults, contribute to the MedDiet’s health benefits, including reduced risks of cardiovascular disease and cancer, as well as improved cognitive health [8,9,10].

Although current evidence suggests a positive association between MedDiet adherence and HRQoL [9,11,12,13], this relationship remains unclear due to the heterogeneity of the samples in terms of age, culture, pathologies, and lifestyles, as well as the different questionnaires used. In the Spanish context, only a few studies have focused specifically on middle-aged adults [14,15,16,17] or older adults [3,18,19]. While these studies consistently used the SF-36 questionnaire to assess HRQoL, none of them used the same tool to evaluate diet patterns, adding further variability to the findings. Despite this, all studies confirmed a positive association between the MedDiet adherence and HRQoL, though none investigated which specific dietary elements might contribute to this relationship. On the other hand, older adults who follow the MedDiet also tend to have higher levels of PA, a confounding variable that is not always controlled in studies but positively influences HRQoL outcomes [13,20]. Despite the benefits of the MedDiet, both the Spanish population and other Mediterranean populations are currently moving away from traditional dietary patterns due to the influence of Westernised diets and lifestyle changes, particularly among younger individuals. In this sense, several studies have reported an increased contribution of animal products and sugars to overall intake. Meat and meat product consumption exceeds recommendations, while the intake of cereals, vegetables, fruits, and legumes falls below recommended levels for the Spanish population [8,13,21,22]. This trend also extends to older adults, who seem to be losing eating habits such as the consumption of extra virgin olive oil, vegetables, fruit, legumes, and nuts [12].

Once the problem approach has been outlined, our initial hypothesis suggests that the progressive decline in the physical component of HRQoL is age-dependent but less pronounced in individuals with higher adherence to the Mediterranean diet. Additionally, this adherence may vary across age groups, not only in overall scores but also in terms of which specific dietary habits are maintained or abandoned with age. Therefore, the aims of the present study are two-fold: (1) to analyse the association between adherence to the Mediterranean diet pattern and the physical component of health-related quality of life across various age groups of active older adults, and (2) to examine the differences in Mediterranean dietary habits between these age groups.

## 2. Materials and Methods

### 2.1. Participants and Study Design

The study utilised a cross-sectional, descriptive, and comparative design. Based on data from the Spanish National Institute of Statistics [23] (https://www.ine.es/ (accessed on 10 October 2022)), a sample size of 274 participants was deemed sufficient with a significance level (α) of 0.05 and a two-tailed confidence interval of 90%. Three inclusion criteria were established: being between 41 and 85 years old, maintaining an active lifestyle in line with WHO physical activity recommendations, and being healthy without any conditions that limit daily activities [8,10,24]. Of the initial 553 participants from various regions of Spain, 33 were excluded because they did not meet the inclusion criteria or did not complete the questionnaires accurately. This resulted in a final sample size of 520 participants, grouped into the following age ranges: 41–50, 51–60, 61–70, and 71–80 years old [24]. Participants were recruited randomly over a three-month period and invited to participate voluntarily after giving informed consent. They were thoroughly informed of the aims of the study and strict confidentiality of their data was ensured. The study adhered to the ethical principles outlined in the Declaration of Helsinki and was approved by the University of Granada’s Research Ethics Committee (code 1230/CEIH/2020, 13 January 2020).

Three inclusion criteria were established: being between 41 and 85 years old, maintaining an active lifestyle in line with WHO physical activity recommendations, and being healthy without any conditions that limit daily activities.

Baseline characteristics of the participants according to age group and sex are shown in Table 1.

### 2.2. Instruments

An ad-hoc questionnaire collected sociodemographic information, including age, gender, weight, and height, which were used to calculate BMI.

Active life was verified using the Spanish version of the Rapid Assessment of Physical Activity Questionnaire (RAPA-Q) [25], a validated and user-friendly tool specifically designed for older adults. This seven-item questionnaire can be answered with “yes” or “no” and effectively determines PA levels. Following the WHO recommendations for cardiovascular health benefits [26], participants engaging in more than 150 min per week of moderate activities or 75 min of vigorous activities were classified as active, while others were categorised as non-active. Only active participants were included in this study.

The Spanish version of the SF-36 questionnaire [27] was used to assess HRQoL. This tool has demonstrated validity and high reliability, and it is widely used in older adult populations [28,29,30]. It is composed of eight domains: physical function (10 items, α = 0.93), physical role (4 items, α = 0.95), bodily pain (2 items, α = 0.87), general health (5 items, α = 0.79), vitality (4 items, α = 0.85), social function (2 items, α = 0.72), emotional role (3 items, α = 0.91), and mental health (5 items, α = 0.85). Each domain is scored on a scale from 0 to 100, with higher scores indicating better health status. Additionally, it provides two summary scores: the physical component (Comp-P) and the mental component (Comp-M), calculated using population-specific weights for Spain [31,32]. The cut-off points for classifying these components into two levels were based on median values from Spanish population norms, adjusted by age and gender [31,32]. In this study, the Cronbach’s α coefficients for the SF-36 exceeded 0.75, indicating satisfactory internal consistency. 

To quantitatively estimate adherence to the MedDiet, the validated Mediterranean Diet Adherence Screener (MEDAS) [33] was used. This questionnaire, originally derived from a validated food frequency questionnaire (FFQ, r = 0.52; *p* < 0.001), was designed to evaluate the impact of the MedDiet on the primary prevention of cardiovascular diseases [33,34,35,36]. MEDAS consists of 14 items, with 12 focusing on the frequency of consumption of key foods (olive oil, wine, fruits, vegetables, fish, legumes, nuts, meat and its derivatives, poultry, butter, pastries, and carbonated/sweetened beverages), and the remaining 2 items assess specific characteristic of the MetDiet, the use of olive oil as cooking fat, and the consumption of chicken [37]. Each affirmative response is awarded one point, with a score of 10 or higher indicating good adherence to the MedDiet, while scores below 10 reflect poor adherence [38]. Based on this, two groups were established, good and poor adherence to the MedDiet.

### 2.3. Statistical Analysis

Statistical analyses were conducted using R statistical software (v 4.1.2., R Core Team, Vienna, Austria). The Kolmogorov–Smirnov test with Lilliefors correction was applied to assess the normality of the variables, while homoscedasticity was checked using Levene’s test. Descriptive statistics are presented as means  ±  standard deviations (SDs) or frequencies. For comparisons of continuous variables between groups, the parametric T-test and one-way ANOVA test were applied, with effect size calculated using Cohen’s d index. The Pearson chi-square test was used to compare categorical variables. Spearman’s rho correlation coefficient was employed for bivariate correlations. The internal reliability of the instruments was evaluated using Cronbach’s Alpha. All *p*-values were two-tailed, with statistical significance set at *p* ≤ 0.05. 

## 3. Results

As shown in Table 1, significant differences (*p* ≤ 0.05) were found when comparing height and BMI across age groups, with the largest differences observed between the 41–50 and 71–80 age groups.

**Figure 1:**
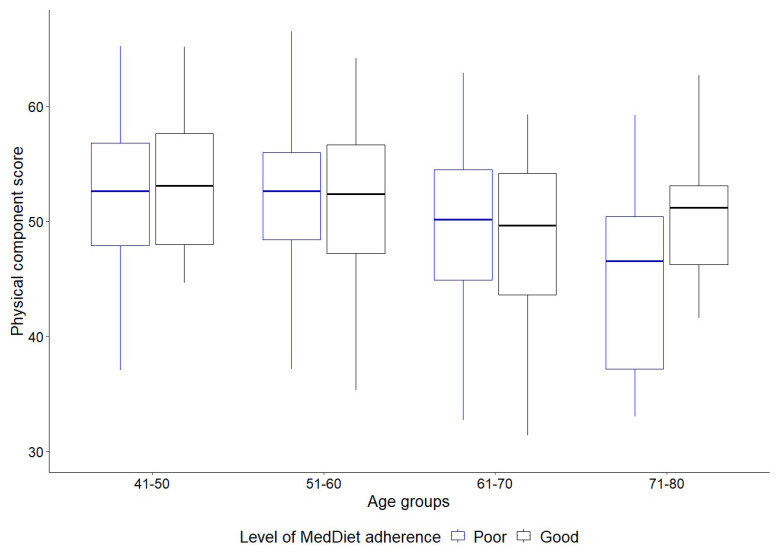
Physical component score by age groups and levels of adherence to MedDiet.

Table 2 and Figure 1 present a comparison of Comp-p scores between age groups in relation to adherence to the Mediterranean diet. The lowest Com-P scores were observed in the 61–70 and 71–80 age groups. With regard to adherence to the MedDiet, significant differences were observed solely in the 71–80 age group (*p* = 0.015, d = 0.74), wherein participants with good adherence exhibited a higher Comp-P (50.10 ± 7.39) than those with poor adherence (44.46 ± 7.73). No significant differences were identified in the remaining age groups.

**Figure 2:**
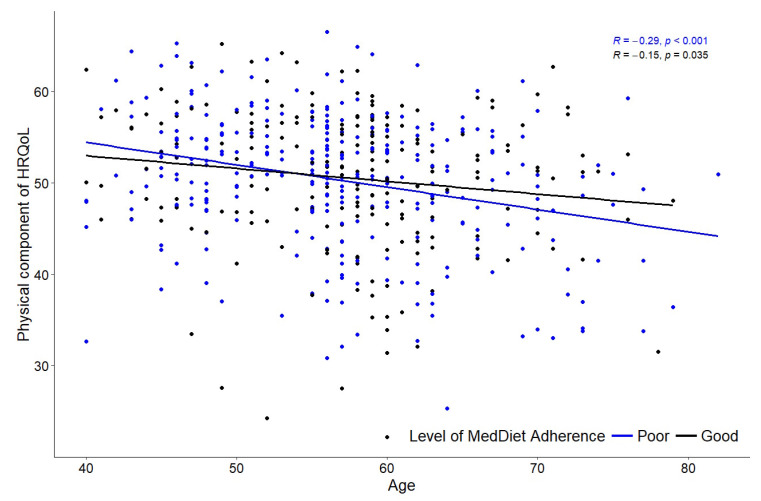
Bivariate correlations between Comp-P and age by levels of adherence to MedDiet.

The analysis of the correlation between the physical component of HRQoL (Com-p), MedDiet adherence, and age is presented in Table 3 and Figure 2. A significant correlation between Com-p and MedDiet adherence was only found in the 71–80 age group (r = 0.367, *p* = 0.014). Between Comp-p and age by MedDiet adherence, both groups presented significant correlations, being higher in poor adherence group (r = −0.29, *p* = 0.035).

The correlation analysis between the physical component of HRQoL (Com-p), MedDiet adherence, and age is summarised in Table 3 and Figure 2. A significant positive correlation between Com-p and MedDiet adherence was found only in the 71–80 age group (r = 0.367, *p* = 0.014). When analysing the correlation between Com-p and age by levels of MedDiet adherence, both groups showed significant negative correlations, with a stronger correlation observed in the poor adherence group (r = −0.29, *p* = 0.001).

**Figure 3:**
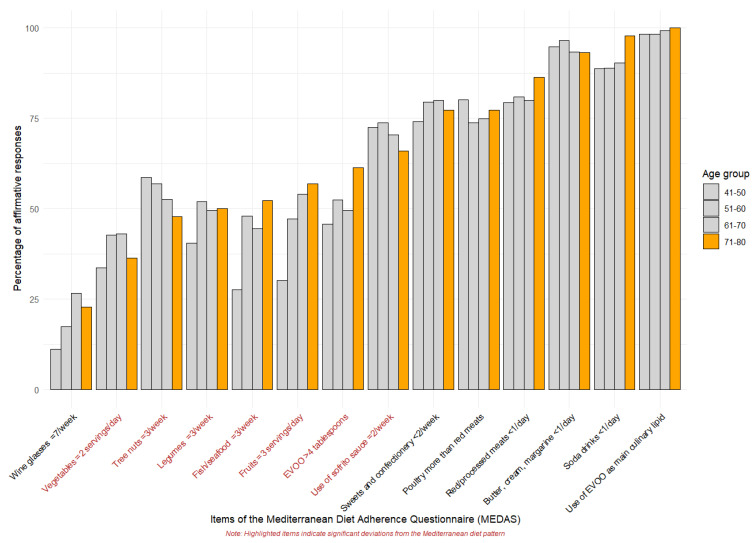
Percentage of affirmative responses to MEDAS questionnaire. In red are items with less than 60% for the 71–80 age group.

Table 4 and Figure 3 present the percentage of positive responses to each MEDAS item, both for the total sample and by age group. In the overall sample, the lowest adherence rates were observed for vegetables (40.07%), fruits (45.74%), tree nuts (47.73%), legumes (50.00%), fish (52.27%), and fruit consumption (56.82%). Significant differences between age groups were found only for fruit (*p* < 0.001) and fish (*p* = 0.002) consumption, with the highest percentages observed in the 71–80 age group (56.82% and 52.27%, respectively).

## 4. Discussion

The aim of this study was to investigate the association between the physical component (Comp-p) of health-related quality of life (HRQoL) and adherence to the Mediterranean diet pattern (MedDiet) across various age groups of active older adults, as well as to examine in detail the differences in Mediterranean dietary habits among these age groups.

The main finding indicated a positive association between MedDiet adherence and Comp-p only in the 71–80 age group. In this group, individuals with good adherence to the MedDiet had higher Comp-p scores, reflecting better physical health. In addition, the negative weak correlation found between Comp-p and age was moderated by MedDiet adherence. These results suggest that greater adherence to the MedDiet is particularly important for active individuals aged 71 years and above, as it may mitigate the age-related decline in physical health, confirming previous studies [20,38,39].

The MedDiet is well known for its benefits in reducing the risk of age-related diseases by decreasing oxidative stress and inflammation [13,38]. Regarding HRQoL, particularly its physical components (Comp-P), previous studies have shown an association with both age [39,40,41,42] and MedDiet adherence in older adults, despite the use of different questionnaires [9,13,39]. Nevertheless, our study found this relation between Comp-P and MedDiet only in participants aged 71 and above and not in those aged 51–70. Additionally, the mean Comp-p scores were very similar across the 41–50, 51–60, and 61–70 age groups. In fact, the negative correlation between the physical component of HRQoL and age was weak among participants with poor MedDiet adherence but almost twice as strong in those with good adherence. 

To understand these discrepancies, it is important to note that participants in our sample maintained an active lifestyle, engaging in a weekly amount of PA in line with WHO recommendations, which was the main methodological difference from previous studies. The positive correlation between HRQoL and PA is well-documented in the literature and may mediate the relationship between HRQoL and MedDiet adherence, potentially slowing the effects of ageing [13].

Muscle mass declines by about 3–8% per decade after the age of 30, with an even greater rate of decline after age 60; by age 70, individuals may expect to lose up to 50% of their muscle mass [43]. This involuntary loss of muscle mass, strength, and function, known as sarcopenia, can lead to functional dependence and disability [44]. Higher levels of PA, including both resistance and aerobic exercise, can counteract sarcopenia, enhancing functional fitness and positively affecting the perception of HRQoL [45]. In summary, despite objective health declines, subjective physical health may be maintained in active older adults during mid-life and even into later life [46].

Westernised dietary patterns, characterised by excessive calorie intake, high consumption of processed foods, and a reduced intake of fruits and vegetables, are being adopted worldwide, including in Mediterranean countries [12]. As a result, MedDiet adherence is being negatively affected, even among older adults [8]. In line with previous studies, we found that although older adults are aware of how to avoid certain Westernised habits such as consuming less processed food, sweets or carbonated beverages, their adherence to the MedDiet is declining due to reduced intake of fresh vegetables, and legumes, fish, fruit and EVOO [11]. This means giving up its many health benefits. Fruits and vegetables are rich in fibre, vitamins, minerals, carotenoids and polyphenols, helping to reduce the risk of type 2 diabetes, cardiovascular disease (CVD) and sarcopenia [38,40]; legumes are a rich source of essential fatty acids, protein, and fibre, helping in the regulation of glycaemic levels and reducing CVD risk [40]; nuts, rich in MUFAs, PUFAs, and antioxidants, improve lipid profiles and slow biologic ageing [38,47]; fish provide omega-3 fatty acids, vitamins, and essential minerals, supporting heart health and reducing oxidative stress [40]; olive oil is a main component in the MedDiet, high in MUFAs, polyphenols, and antioxidants, protecting against CVD, improving cholesterol levels, and reducing inflammation [47].

The lack of consumption of these foods is not necessarily due to a lack of education or habits, as is often the case in younger populations. Rather, these components of the Mediterranean diet are becoming increasingly expensive, making them less accessible to older adults with lower incomes and mainly consumed by those in the higher socioeconomic group [15,40]. This problem is further compounded by reduced mobility, which hinders their ability to purchase and transport fresh produce on a daily basis [15].

Limitations and perspective. Notwithstanding the above, the lowest values in Comp-P in individuals over 70 could also be a result of poor nutrition during their childhood and adolescence, rather than current dietary habits, as this population was born and grew up during the post-war period, particularly during the so-called ‘hunger years,’ which lasted until the late 1960s [48]. This generation experienced inadequate nutrition, which likely affected their physical development and may still be influencing their current health. Moreover, this generation began working at an earlier age under more demanding physical conditions, mainly in the primary sector. This hypothesis is supported by the fact that the shortest participants in our study were also the oldest. Therefore, results related to this generation should be interpreted with caution.

Given the cross-sectional design of the study, establishing causality between the variables analysed is not possible. Longitudinal data are needed for a more detailed and comprehensive understanding of these relationships. In addition, the absence of objective measures to assess the variables in this study necessitates a cautious interpretation of the results. Finally, the sample used in this study, consisting entirely of individuals engaged in physical activities, makes it difficult to compare the results and generalise the findings. Further prospective studies are needed to confirm these results, incorporating other factors such as PA levels and socioeconomic status.

## 5. Conclusions

In individuals aged 71–80, lower adherence to the Mediterranean diet is associated with a decline in self-perceived physical health, mainly due to the reduced consumption of fresh vegetables, legumes, fish, and fruit. These findings highlight the importance of these foods, provide valuable insights for tailored dietary interventions, and highlight the critical role of maintaining adherence to the Mediterranean diet, especially in later life, to mitigate age-related declines in physical health and support overall well-being. Given the complexity of the relationship between diet and health, promoting lifelong adherence to the Mediterranean dietary patterns—particularly regular consumption of these key food groups and preserving traditional recipes from this cultural heritage—along with physical activity should be prioritised in older adults to preserve functional fitness and quality of life.

## Figures and Tables

**Table 1:** Sample characteristics according to age groups and sex.

*n* = 520		41–50 (*n* = 116)			51–60 (*n* = 225)			61–70 (*n* = 135)			71–80 (*n* = 44)			
		M	W	*p_sex_*	M	W	*p_sex_*	M	W	*p_sex_*	M	W	*p_sex_*	*P_group_*
Distribution	*n*	66	50	-	173	52	-	102	33	-	28	16	-	-
	%	56.89	43.10	0.167	76.88	23.11	0.001	75.55	24.44	0.001	63.63	36.36	0.006	0.005
Age (years)	Mean	45.58	45.50	0.952	55.69	55.29	0.461	63.21	63.39	0.688	73.86	71.81	0.007	0.001
	SD	2.55	2.74	-	2.68	2.87	-	2.73	2.83	-	2.90	2.83	-	-
Height (m)	Mean	1.77	1.64	0.001	1.74	1.60	0.001	1.73	1.60	0.001	1.70	1.58	0.001	0.001
	SD	7.66	7.31	-	7.51	9.60	-	7.77	6.31	-	7.21	4.76	-	-
Weight (kg)	Mean	82.69	62.8	0.001	81.46	62.52	0.001	79.69	67.1	0.001	75.78	69.09	0.100	0.149
	SD	12.55	15.13	-	12.13	9.19	-	10.82	14.64	-	8.00	12.31	-	-
BMI (kg/m^2^)	Mean	26.31	23.30	0.001	26.72	24.52	0.001	26.69	26.00	0.175	26.23	27.71	0.537	0.002
	SD	4.19	5.46	-	4.36	4.23	-	4.33	5.55	-	3.22	5.34	-	-

SD: standard deviation; M: man: W: woman.

**Table 2:** Physical component score by levels of adherence to MedDiet and age groups.

Age Group	Good Adherence MedDiet		Poor Adherence MedDiet		Sig.	Effect Size	
	Distribution *n*, %	Comp-p Score M(SD)	Distribution *n*, %	Comp-p Score M(SD)	*p*	*d*	CI
41–50	37, 31.90	52.30(7.72)	79, 68.10	52.39(6.81)	0.868	0.01	(−0.38, 0.40)
51–60	98, 43.96	51.42(7.36)	127, 56.44	51.35(6.79)	0.750	0.01	(−0.27, 0.25)
61–70	57, 42.22	48.06(7.94)	78, 57.78	48.78(7.26)	0.733	0.09	(−0.25, 0.44)
71–80	18, 40.91	50.10(7.39)	26, 59.09	44.46(7.73)	0.015	0.74	(0.12, 1.36)

M: mean; SD: standard deviation; *d*: Cohen’s d; CI: confidence interval.

**Table 3:** Bivariate correlation of Comp-p with MedDiet and age by groups.

Comp-P and MedDiet by Age Groups			
Group	r	CI	*p*
41–50	−0.014	(−0.19, 0.12)	0.876
51–60	−0.007	(−0.22, −0.04)	0.912
61–70	−0.028	(−0.20, 0.14)	0.740
71–80	0.367	(0.06, 0.65)	0.014
**Comp-P and Age by MedDiet Adherence Levels**			
**Group**	**r**	**CI**	***p***
Poor MedDiet	−0.29	(−0.39, −0.18)	0.001
Good MedDiet	−0.15	(−0.28, −0.01)	0.035

**Table 4:** Percentage of affirmative responses to MEDAS Questionnaire.

	MEDAS Questionnaire	All	Age Group				Sig.	Effect Size
			41–70	51–60	61–70	71–80		
Item	Question	% Yes	% Yes	% Yes	% Yes	% Yes	*P*	Cramer´s V
1	Use of EVOO as the main culinary lipid	98.48	98.28	98.22	99.26	100	0.556	0.072
2	EVOO >4 tablespoons	50.47	45.69	52.44	49.63	61.36	0.149	0.115
3	Vegetables ≥2 servings/day	40.07	33.62	42.67	42.96	36.36	0.434	0.083
4	Fruits ≥3 servings/day	45.74	30.17	47.11	54.07	56.82	0.001	0.208
5	Red/processed meats <1/day	80.71	79.31	80.89	80.00	86.36	0.558	0.072
6	Butter, cream, margarine <1/day	95.08	94.83	96.44	93.33	93.18	0.722	0.058
7	Soda drinks <1/day	89.79	88.79	88.89	90.37	97.73	0.074	0.131
8	Wine glasses ≥7/week	18.90	40.52	52.00	49.63	50.00	0.369	0.089
9	Legumes ≥3/week	48.58	40.52	52.00	49.63	50.00	0.369	0.089
10	Fish/seafood ≥3/week	42.91	27.59	48.00	44.44	52.27	0.002	0.189
11	Commercial sweets <2/week	78.07	74.14	79.56	80.00	77.27	0.741	0.056
12	Tree nuts ≥3/week	55.38	58.62	56.89	52.59	47.73	0.414	0.085
13	Poultry more than red meats	75.42	80.17	73.78	74.81	77.27	0.716	0.058
14	Use of sofrito sauce ≥2/week	71.83	72.41	73.78	70.37	65.91	0.635	0.065

Note: EVOO (extra virgin olive oil); MEDAS (Mediterranean Diet Adherence Screener).

## Data Availability

There are restrictions on the availability of data for this trial due to the signed consent agreements around data sharing, which only allow access to external researchers for studies following the project’s purposes. Requestors wishing to access the trial data used in this study can make a request to mariscal@ugr.es.
